# Early passive mobilization increases vascular reactivity response in critical patients with sepsis: a quasi-experimental study

**DOI:** 10.5935/0103-507X.20220132-en

**Published:** 2022

**Authors:** Tamara Rodrigues da Silva Destro, Thaís Marina Pires de Campos Biazon, Henrique Pott-Junior, Flávia Cristina Rossi Caruso, Daniela Kuguimoto Andaku, Naiara Molina Garcia, José Carlos Bonjorno-Junior, Audrey Borghi-Silva, Débora Mayumi de Oliveira Kawakami, Viviane Castello-Simões, Renata Gonçalves Mendes

**Affiliations:** 1 Department of Physical Therapy, Universidade Federal de São Carlos - São Carlos (SP), Brazil.; 2 Department of Medicine, Universidade Federal de São Carlos - São Carlos (SP), Brazil.

**Keywords:** Sepsis, Endothelium, vascular, Exercise, Hospitalization, Inpatient

## Abstract

**Objective:**

To investigate the influence of a passive mobilization session on endothelial function in patients with sepsis.

**Methods:**

This was a quasi-experimental double-blind and single-arm study with a pre- and postintervention design. Twenty-five patients with a diagnosis of sepsis who were hospitalized in the intensive care unit were included. Endothelial function was assessed at baseline (preintervention) and immediately postintervention by brachial artery ultrasonography. Flow mediated dilatation, peak blood flow velocity and peak shear rate were obtained. Passive mobilization consisted of bilateral mobilization (ankles, knees, hips, wrists, elbows and shoulders), with three sets of ten repetitions each, totaling 15 minutes.

**Results:**

After mobilization, we found increased vascular reactivity function compared to preintervention: absolute flow-mediated dilatation (0.57mm ± 0.22 *versus* 0.17mm ± 0.31; p < 0.001) and relative flow-mediated dilatation (17.1% ± 8.25 *versus* 5.08% ± 9.16; p < 0.001). Reactive hyperemia peak flow (71.8cm/s ± 29.3 *versus* 95.3cm/s ± 32.2; p < 0.001) and shear rate (211s ± 113 *versus* 288s ± 144; p < 0.001) were also increased.

**Conclusion:**

A passive mobilization session increases endothelial function in critical patients with sepsis. Future studies should investigate whether a mobilization program can be applied as a beneficial intervention for clinical improvement of endothelial function in patients hospitalized due to sepsis.

## INTRODUCTION

A healthy endothelium has been described as the key regulator of vascular homeostasis.^(1)^ Among other properties, endothelial cells produce a variety of vasoregulatory substances, including nitric oxide.^(1)^ Under normal conditions, endothelial nitric oxide synthase (eNOS) produces nitric oxide from L-arginine in response to physical stimuli, leading to vasodilatation.^(2)^

However, in sepsis,^(3,4)^ as a maladaptive response, the bioavailability of eNOS is impaired, and dysregulated inducible nitric oxide (NO) synthase (iNOS) isoform activity has been observed at advanced stages. This alteration culminates in organism-wide vasodilatation, increased vascular permeability and diffuse alterations in microvascular perfusion.^(3)^ Recently, Dolmatova et al.^(5)^ described in a detailed review the effects of sepsis on the endothelium and its clinical implications.

In general, the endothelium contributes to local control of infection (vasodilation, permeability and coagulation) to allow defense cells to reach the infection site and prevent dissemination.^(5)^ However, in the context of systemic activation, in response to cytokine production, the endothelium expresses adhesion molecules and produces vasoactive compounds, inflammatory cytokines, and chemoattractants, resulting in microvascular thrombosis, capillary permeability, hypotension, tissue hypoxia, and ultimately tissue damage.

In other pathological contexts of endothelial dysfunction, several interventions have been researched in an effort to prevent or minimize this dysfunction, including physical exercises.^(6,7)^ Physical exercise has been shown to stimulate endothelial cells by increasing shear stress.^(8,9)^ More specifically, the local blood flow augmentation, in response to physical exercise, promotes an increase in the frictional force on the vessel wall, triggering the activation of eNOS by endothelial cells, NO release and a vasodilation response. Due to an inability to collaborate, passive exercises are the most widely used strategy to mobilize critically ill patients.

Although physical exercise has been proven to be a stimulus to promote an increase in vascular function, there is no evidence that physical exercise increases function in a dysfunctional endothelium, as found in patients diagnosed with sepsis. Therefore, the aim of this study was to investigate the influence of a passive mobilization (PM) session on endothelial function in patients with sepsis. We hypothesized that a PM session may stimulate the physiological eNOS pathway and beneficially influence the vascular reactivity response (VRR) postintervention in patients with sepsis. This knowledge may be a precursor to future investigations into the potential of mobilization as a supplement to endothelial dysfunction-targeted therapies in these patients.

## METHODS

This is a quasi-experimental double-blind and single-arm study with a pre- and postintervention design. This study was conducted in an intensive care unit (ICU) in São Carlos, SP, Brazil between 2015 and 2017 and was approved by the local Research Ethics Board (CAAE: 58405916.4.0000.5504, protocol number: 2.363.397), conducted in accordance with the ethical principles of the Declaration of Helsinki, and all subjects and/or responsible agents gave written informed consent. The present study was registered in the *Registro Brasileiro de Ensaios Clínicos* - REBEC (U1111-1215-9989).

Patients were enrolled if they met the following criteria: age between 18 and 70 years, diagnosed with sepsis^(10)^ and within the first 24 - 48 hours of the onset of the disease, on invasive mechanical ventilation and having Richmond Agitation-Sedation Scale score of -5. Exclusion criteria included medium-high dose of norepinephrine (≥ 0.5mcg/kg/minute), coagulopathy (prothrombin time > 2.5 times the normative values; activated partial thromboplastin time > 2 times the normal; or platelet count ≤ 50.000/µL), anticoagulant therapy with heparin intravenous infusion ≥ 2UI/mL, persistent arrhythmias, recent myocardial infarction (< 6 months), presence of pacemakers, intracranial hypertension, body mass index greater than 40kg/m^2^, cancer chemotherapy, unconsolidated fracture, pregnancy, anemia, amputation, deep vein thrombosis or phlebitis, musculoskeletal deformity, and compartment syndrome.^(11)^

To classify the severity of disease, the Acute Physiology and Chronic Health Evaluation (APACHE II) score was used, which uses a point score based upon initial values of 12 routine physiologic measurements, age, and previous health status to provide a general measure of severity of disease;^(12)^ in addition, the Sequential Organ Failure Assessment (SOFA) score was used to track the performance of the body’s organic systems (neurological, blood, hepatic, renal and blood pressure/hemodynamics) during the patients’ stay in the ICU.^(13)^ Sample characterization data were obtained on the day of assessment, and medications were assessed at the time of data collection.

### Intervention - passive mobilization

**Figure 1:**
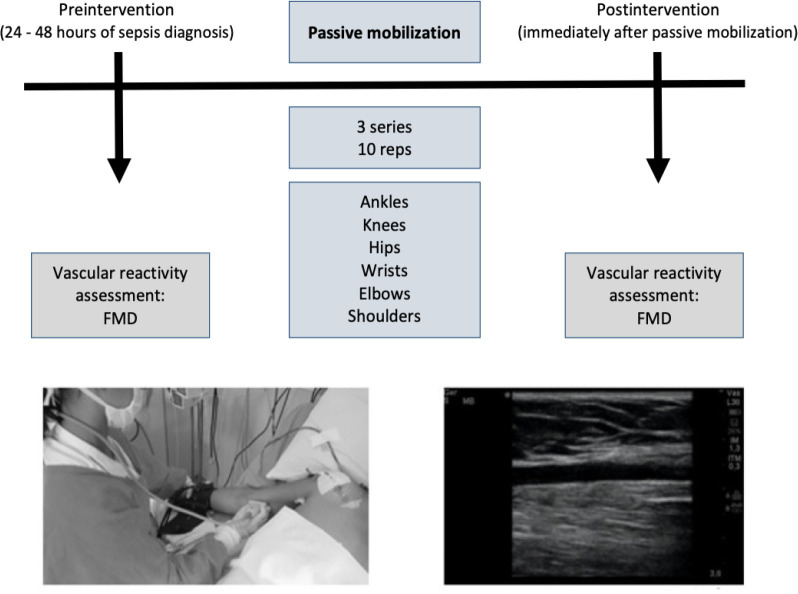
Summary of the experimental protocol.

Patients underwent the experimental protocol within 24 - 48 hours after admission to the ICU. The protocol included bilateral mobilization of the ankles, knees, hips, wrists, elbows and shoulders. Mobilizations were carried out throughout the range of movement (dorsiflexion and plantar flexion; flexion and extension of knees and hips; flexion and extension of wrists, elbows and shoulder). Bilateral mobilization of the ankles, wrists and elbows was performed simultaneously by a single physiotherapist, whereas mobilization of the knees, hips and shoulders was performed alternately. Three sets of ten repetitions were performed for each joint with a resting interval of 15 seconds between sets and 30 seconds to change joints. The frequency of the movements was maintained using a digital metronome (KORG, Inagi, TK, Japan)^(11)^ with a count of 64 beeps per minute, with a beep for flexion and a beep for extension. Each joint was mobilized for approximately 1 minute, totaling 6 minutes of joint movement and 12 minutes of the experimental protocol. Figure 1 summarizes the experimental protocol. Prior to mobilization, patients in this study were screened using the safety criteria described: body temperature from 36.5 - 37.5°C, systolic blood pressure from 100 - 150mmHg, diastolic blood pressure from 60 - 100mmHg, peripheral oxygen saturation > 90%; respiratory rate < 25 breaths/minute, heart rate from 60 - 140 beats/minute and fraction of inspired oxygen < 60%. The interruption criteria of the PM protocol were as follows: appearance of arrhythmias, peripheral oxygen saturation < 90%, presence of signs of respiratory distress, ± 20bpm/min heart rate changes and reduction or increase in mean arterial pressure of 20mmHg.

FMD - flow-mediated dilation.

### Study assessments and outcomes

To ensure blinding of the study investigators, a blinded physiotherapist was assigned to apply the experimental protocol, whereas another blinded professional was responsible for carrying out the evaluations and analysis of the coded data. Data analysis was performed by a blinded statistical expert. Patients were unaware of the intervention due to unconsciousness.

The primary study outcome was the mean change in VRR from baseline (preintervention) to immediately postintervention. The vascular reactivity response was assessed by flow-mediated dilation (FMD) of the brachial artery. Measurements were obtained with patients in the supine position (15 minutes) with high-resolution ultrasonography (M-Turbo, Fujifilm Sonosite, Bothell, WA, United States) near the right antecubital fossa, with the arm abducted 80 degrees from the body. Baseline measurements were performed, and the probe position was marked. Afterward, the right brachial artery was occluded by inflating a cuff placed on the forearm to 240mmHg for 5 minutes. Blood flow was recorded prior to and immediately after cuff release for 20 seconds. During the reactive hyperemia phase, continuous B-mode images were collected for 3 minutes after cuff release.^(14)^ Digital recordings were later analyzed using Brachial Imager software (Medical Imaging, Iowa City, IA, USA). The coefficient of variation for diameter was 1.8%, and the coefficient of variation for FMD in consecutive scans was 13.6%.^(1)^ Relative FMD was calculated as = (reactive hyperemia diameter - baseline diameter)/baseline diameter x 100; absolute FMD was calculated as = reactive hyperemia diameter - baseline diameter.^(15)^ The FMD response to PM was calculated as follows: relative FMD immediately postintervention - relative FMD baseline (preintervention). The peak shear rate was used to estimate brachial artery shear stress and was calculated as reactive hyperemia peak blood flow velocity/baseline diameter. Normalized FMD for shear rate was calculated as absolute FMD/peak shear rate.^(14,15)^

### Sample size and minimal clinically important difference

A sample size of 33 patients was estimated a priori to ensure a statistical power of 80% at a 5% significance level to detect at least a medium effect size for the intervention based on Cohen’s description of effect sizes.^(16)^ The medium effect size was considered the minimal clinically important difference (MCID)^(17)^ for the change in mean FMD% response from preintervention to postintervention.

The a priori MCID was established as an increase of 9,28% in FMD response from baseline, based on the distribution method, i.e., MCID ≥ 0.5 x standard deviation^(18)^ of baseline FMD% response. To obtain the mean baseline FMD% values, we used data reported by Bonjorno Junior et al.^(19)^ As the abovementioned authors presented data for two different groups separately, we calculated the pooled standard deviation as the weighted average of two standard deviations from the two groups.^(19)^

### Statistical analysis

Continuous data are presented as the mean ± standard deviation according to the Shapiro-Wilk test of normality. Categorical variables are presented as counts and percentages. Continuous data were analyzed using linear mixed-effects models fit by residual maximum likelihood. “Subject” was used as a random effect. The fixed effect was “Timing” (pre/postintervention). Statistical significance was assessed at a two-sided p < 0.05. All analyses were conducted using R 3.6.2 (The R Project for Statistical Computing, 2019®) in R Studio 1.3.443 (RStudio Inc., Boston, MA, USA).

## RESULTS

**Figure 2:**
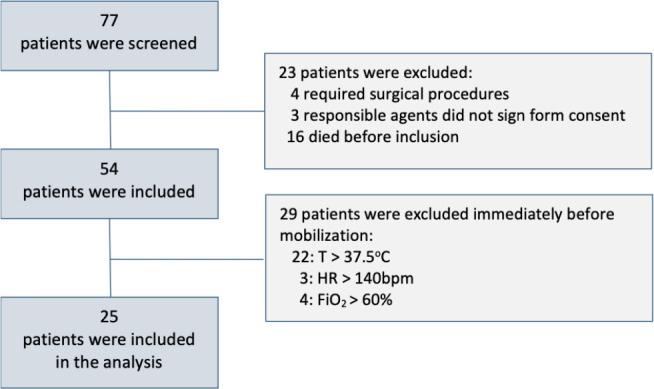
Flowchart of the study population.

Initially, 77 patients were screened through medical recordings for eligibility; however, before effective inclusion (24 - 48 hours after the onset of the disease), 23 patients did not meet all inclusion criteria. At the time of bedside assessments, 29 were excluded due to safety criteria, and 25 patients were included in the final sample, which was adequate due to the larger effect size obtained (Figure 2). The included patients were predominantly females (56%) with a mean age of 56 ± 13 years. The mean times from hospital admission to sepsis diagnosis and to intensive care unit admission were 7.5 ± 3.5 and 101.8 ± 139.7 hours, respectively (Table 1).

T- temperature; HR - heart rate; FiO_2_ - oxygen inspired fraction.

**Table 1:** Characteristics of patients within 24-48 hours of admission to the intensive care unit

Variables	Septic patients (n = 25)
General characteristics	
Age (years)	56 ± 13
Male	11 (44)
Weight (kg)	71.6 ± 9.6
BMI (kg/m^2^)	27.5 ± 3.5
Time points	
Hospital admission until sepsis diagnosis (hours)	7.5 ± 3.5
Hospital admission until ICU (hours)	101.8 ± 139.7
Sepsis origin	
Pulmonary	16 (64)
Abdominal	4 (16)
Urinary tract	3 (12)
Central nervous system	1 (4)
Skin and soft tissue	1 (4)
Clinical data	
Temperature (°C)	37.0 ± 0.9
Heart rate (bpm)	107.3 ± 17.7
Systolic blood pressure (mmHg)	121.0 ± 15.6
Diastolic blood pressure (mmHg)	72.7 ± 12.0
SpO_2_ (%)	96.9 ± 1.9
Respiratory rate (rpm)	20.3 ± 5.1
SOFA	11.1 ± 2.9
APACHE II	30.6 ± 5.9
APACHE II mortality (%)	66.6 ± 18.9
Laboratory data	
CRP (mg/dL)	14.2 ± 9.4
Hemoglobin (g/dL)	10.1 ± 2.3
Leukocytes (x10^3^/mm^3^)	20.6 ± 11.3
Platelets (x10^3^/mm^3^)	270.3 ± 145.3
Log lactate (mmol/L)	2.6 ± 1.1
Creatinine (mg/dL)	2.4 ± 1.5
Total Bilirubin (mg/dL)	1.0 ± 1.1
Urine output (mL/24 hours)	1147.8 ± 1378.6
Vasoactive drugs	
Dobutamine	1 (5)
Norepinephrine	13 (68)
Norepinephrine/dobutamine,	5 (26)
Dosage of vasoactive drugs (µg/kg/mn)	
Dobutamine	0.31 ± 0.23
Norepinephrine	0.6
Norepinephrine/dobutamine	0.38 ± 0.46/1.06 ± 0.88

BMI - body mass index; ICU - intensive care unit; SpO2 - peripheral oxygen saturation; SOFA - Sequential Organ Failure Assessment score; APACHE II - Acute Physiology and Chronic Health Evaluation II; CRP - C-reactive protein. Results expressed as the mean ± standard deviation or n (%).

Table 2 summarizes endothelial function data pre- and postintervention. We found an increased vascular reactivity response in the postintervention: absolute and relative FMD (p < 0.001). More than half (52%) of the patients presented an MCID for the change in mean FMD% response from preintervention to postintervention when comparing the results with external data for the mean baseline FMD% response. However, this proportion increased to 80% (20/25) when considering the values for the study population, i.e., the standard deviation of the baseline FMD% response. The reactive hyperemia peak blood flow velocity and shear rate were also increased compared to the preintervention values (p < 0.001).

**Table 2:** Endothelial function data pre- and postintervention of critical septic patients

Variables	Preintervention	Postintervention	Mean difference (95%CI)	p value
Baseline diameter (mm)	3.68 ± 0.72	3.58 ± 0.76	-0.10 (-0.50 - 0.31)	0.1
Reactive hyperemia diameter (mm)	3.85 ± 0.75	4.15 ± 0.76	0.30 (-0.11 - 0.72)	< 0.001
Relative FMD (%)	5.08 ± 9.16	17.1 ± 8.25	12.00 (7.32 - 16.80)	< 0.001
Absolute FMD (mm)	0.17 ± 0.31	0.57 ± 0.22	0.40 (0.26 - 0.55)	< 0.001
Normalized FMD (mm/s)	0.001 ± 0.003	0.002 ± 0.001	0.001 (0.000 - 0.002)	0.05
Peak shear rate (s)	211 ± 113	288 ± 144	77.67 (7.57 - 151.63)	< 0.001
Baseline peak blood flow velocity (cm/s)	59.7 ± 29.4	68.2 ± 26.1	8.5 (0.16 - 15.8)	0.05
Reactive hyperemia peak blood flow velocity (cm/s)	71.8 ± 29.3	95.3 ± 32.2	23.47 (13.6 - 32.4)	< 0.001

SE - standard error; APACHE II - Acute Physiology and Chronic Health Evaluation score II; SD - standard deviation.

**Figure 3:**
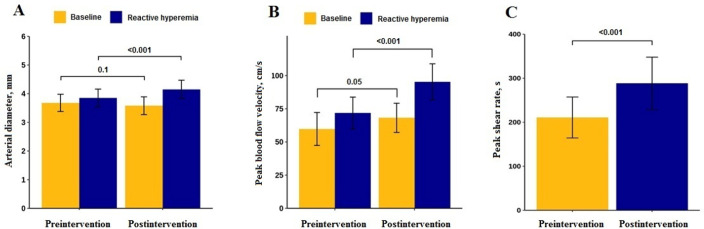
Mean changes from baseline to reactive hyperemia at each time point. (A) Arterial diameter; (B) Peak blood flow velocity; and (C) Peak shear rate.

**Figure 4:**
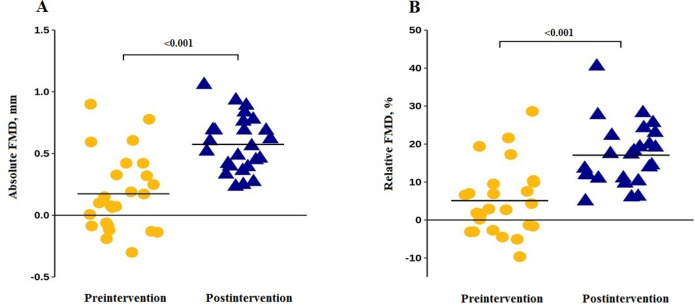
Individual changes in the absolute (A) and relative (B) flow-mediated dilation from pre- to postintervention.

Figure 3 shows the mean changes in arterial diameter, peak blood flow velocity and peak shear rate from baseline to reactive hyperemia at each time point. As shown in figure 3A, the postintervention arterial diameter presented higher values of reactive hyperemia than the preintervention arterial diameter (p < 0.001). In relation to peak blood flow velocity, higher values of reactive hyperemia were observed postintervention compared to preintervention (p < 0.001) (Figure 3B). In addition, we observed that the peak shear rate presented higher values postintervention than preintervention (p < 0.001) (Figure 3C). In addition, we observed in figure 4A and figure 4B individual changes in the absolute and relative FMD (respectively) from pre- to postintervention. We reported the difference in FMD in absolute and relative postintervention compared to preintervention (p < 0.001).

95%CI - 95% confidence interval; FMD - flow-mediated dilation. Results expressed as the mean ± standard deviation.

FMD - flow-mediated dilation.

Considering that more severe cases could also have a direct relationship with lowering the VRR, we performed a multivariate adjusted linear mixed-effects regression to model the relative FMD as a function of “Timing” (pre/postintervention) and its interaction term with APACHE II score. “Subject” was used as a random effect. Table 3 shows that the intervention remained statistically significant after correcting the APACHE II score.

**Table 3:** Linear mixed effects model summary

	Model 1			Model 2			Model 3		
Fixed effects	Estimate	SE	p value	Estimate	SE	p value	Estimate	SE	p value
Intercept	11.075	1.491	< 0.001	5.078	1.744	0.005	8.716	7.633	0.2
Intervention				11.995	2.093	< 0.001	11.995	2.093	< 0.001
APACHE II							-0.119	0.243	0.6
Random effect	Variance	SD		Variance	SD		Variance	SD	
Subject	0.0	0.0		21.23	4.608		22.82	4.777	
Residual	111.2	10.54		54.77	7.401		54.77	7.401	

## DISCUSSION

This study investigated the influence of a PM session on endothelial function in patients with sepsis. The main finding of this study was an increased FMD, peak blood flow velocity and peak shear rate immediately after mobilization. These findings confirmed our hypothesis that the blood flow shift promoted by the mobilization stimulus is able to increase VRR acutely in critical patients with sepsis. These results emphasize a potential contribution of rehabilitation to endothelial function-targeted therapies in patients with sepsis.

Sepsis affects practically all aspects of endothelial function, which remains one of the most compelling targets for therapeutic development.^(5)^ Flow-mediated dilation, a gold standard measure, is one of the most noninvasive promising methods under scientific investigation.^(14)^ The results of previous studies demonstrated a reduced FMD in septic patients,^(19-21)^ in accordance with our results. A previous study^(20)^ observed FMD of 4.8% and 10.7% assessed up to 72 hours after admission to the intensive care unit. A recent study^(19)^ also demonstrated a reduced relative FMD of -2.5% and 10.1% for nonsurviving patients and surviving patients, respectively, at 24 - 48 hours after the sepsis diagnosis, and in our study, we found a mean FMD of 5.08% within 24 - 48 hours. Divergences in basal values can be explained by different comorbidities, medications, individual physiological responses and different time points of assessments in the course of disease.

Additionally, a previous study^(22)^ noted the effects of different types of exercises and times of assessment, and a biphasic response behavior was proposed depending on the patient’s condition, stimulus and time point of the assessment after exercise. For example, when performing high-intensity exercise, it is expected that there will be a reduction in arterial diameter immediately after exercise due to oxidative stress and then dilation.

As mentioned above, patients with sepsis already exhibit dysfunctional endothelium with lower baseline FMD, supporting a crucial role of the endothelium in the pathogenesis of the disease.^(3,23)^ More specifically, during the systemic inflammation of sepsis, iNOS enzyme is upregulated by endotoxins, interferons, tumor necrosis factor alpha (TNF-alpha) and other proinflammatory mediators, which also promote downregulation of e-NOS.^(3,24)^ Consequently, there is a vascular reactivity dysfunction^(25,26)^ leading to a vascular vasodilation insensitivity to the shear stress of blood flow on the endothelial wall.^(27,28)^

Regarding additional methods to assess endothelial function, a group of authors have previously published a passive mobilization of the lower limb as the hyperemic stimulus to VRR,^(29,30)^ in contrast with the FMD method in which the occlusion of the arterial bed is applied to reach the hyperemic stimulus. PM has also been commonly used as a therapeutic strategy for critically ill patients in a comatose state^(31)^ who are unable to collaborate with a more active mobilization rehabilitation strategy. Despite this, the effect of PM as a physical therapy intervention on the VRR of critically ill septic patients is not well known and an interesting field of investigation.

In the present study, we found an improvement in the FMD response, which is a marker of endothelial function, after a PM session stimulus. Therefore, we suggest that the hyperemic stimulus of the FMD associated with the blood flow augmentation stimulated by a PM session was able to promote a better reactive vasodilation response.

More specifically, nitric oxide release by eNOS, mediated by the calcium-calmodulin cascade, is stimulated by shear stress in the endothelium wall, which promotes the vasodilator response.^(27,28)^ According to the current study, a session of PM was apparently able to increase systemic blood flow and consequently shear stress, contributing to the release of nitric oxide by eNOS and, therefore, to physiological arterial vasodilation even in conjunction with the reduction of vascular tone due to the systemic inflammation of septic patients.

In addition, in our study, a significant increase was observed in the peak blood flow velocity in reactive hyperemia in the postintervention compared to the pre-intervention, as well as for the peak shear rate, which presented higher values in the postintervention compared to preintervention. Previously, authors^(23)^ demonstrated lower peak blood flow velocity in sepsis compared to healthy individuals; however, no stimulus such as PM was applied.

In a complementary way, we investigated whether those more severe cases would have a relationship with lowering the VRR; however, the intervention remained statistically significant in respect to the FMD results after correcting for the APACHE II score. Therefore, the high FMD value and the increase in peak blood flow velocity and shear rate after PM in our study can be considered indicators of acute positive clinical response of septic patients even after a single session of PM. Finally, given the scientific evidence of the positive effect of a PM session on the VRR of septic patients and indications of possible acute improvement in the clinical evolution of these patients, we understand that this knowledge can contribute in part to high-level recommendations to study endothelial function protective or restorative modalities in sepsis.

There are some limitations to our study that should be mentioned. First, we did not assess endothelial-independent dilatation to nitroglycerin as a complement to our results. Second, a control group for future comparisons would be interesting to determine differences between groups and to assess the effect of PM on VRR isolated from the hyperemic effect of the release of occlusion pressure during FMD. Third, there is a need for greater homogenization of the sample, as well as the creation of balanced subgroups with and without vasoactive drugs and classified according to the time of diagnosis of sepsis.

## CONCLUSION

A passive mobilization session was able to increase the endothelial function response in critical patients with sepsis. Future studies should investigate whether a mobilization program can be applied as a complementary therapeutic method of endothelial function-targeted therapies.
